# Microtubule-associated protein tau in murine kidney: role in podocyte architecture

**DOI:** 10.1007/s00018-021-04106-z

**Published:** 2022-01-27

**Authors:** Laura Vallés-Saiz, Rocio Peinado-Cahuchola, Jesús Ávila, Félix Hernández

**Affiliations:** Centro de Biología Molecular “Severo Ochoa”, CSIC/UAM, Universidad Autónoma de Madrid, Cantoblanco, 28049 Madrid, Spain

**Keywords:** Kidney, Tau, Podocyte, Glomerular damage

## Abstract

Tau is a cytoskeletal protein that is expressed mainly in neurons and is involved in several cellular processes, such as microtubule stabilization, axonal maintenance, and transport. Altered tau metabolism is related to different tauopathies being the most important Alzheimer’s disease where aberrant hyperphosphorylated and aggregated tau is found in the central nervous system. Here, we have analyzed that function in kidney by using tau knockout mice generated by integrating GFP-encoding cDNA into exon 1 of *MAPT* (here referred to as Tau^GFP/GFP^). IVIS Lumina from PerkinElmer demonstrated GFP expression in the kidney. We then demonstrated by qPCR that the main tau isoform in the kidney is Tau4R. The GFP reporter allowed us to demonstrate that tau is found in the glomeruli of the renal cortex, and specifically in podocytes. This was further confirmed by immunohistochemistry. Tau^G*FP/GFP*^ mice present a podocyte cytoskeleton more dynamic as they contain higher levels of detyrosinated tubulin than wild-type mice. In addition, transmission electron microscopy studies demonstrated glomerular damage with a decrease in urinary creatinine. Our results prove that tau has an important role in kidney metabolism under normal physiological conditions.

**Supplementary Information:**

The online version contains supplementary material available at 10.1007/s00018-021-04106-z.

## Introduction

Tau is a microtubule-associated protein that plays a critical role in the pathogenesis of several disorders in the nervous system collectively known as tauopathies [1–3]. Alzheimer’s disease is the most prevalent tauopathy and the main cause of dementia among older adults. In this disease, intracellular tau protein forms filamentous structures of aggregated protein which are associated with neuronal death. Tau in polymerized or monomeric form is released into the extracellular space through physiological and pathological mechanisms and extracellular tau can be toxic for neighboring cells [4]. This effect may contribute to the progression of a number of neurodegenerative diseases [4, 5].

Tau promotes the polymerization and assembly of neuronal microtubules [6, 7], although additional roles in the nervous system have been suggested taking into account its presence in oligodendrocytes [8, 9], other glial cells [10], and the description of new interaction partners and different subcellular localizations for tau [11–13]. Human Tau protein isoforms are expressed from a unique gene located at chromosome 17 and have 16 exons [14]. Six different isoforms of tau are expressed in the adult human central nervous system via alternative splicing of the *MAPT* gene. Inclusion of exons 2 and 3 yields tau isoforms with 0, 1, or 2N-terminal inserts, whereas exclusion or inclusion of exon 10 leads to expression of tau isoforms with three (3R) or four (4R) microtubule-binding repeats [15, 16]. The expression of these Tau isoforms is regulated in mice during development. Tau 3R isoforms are present in early stages of development, while Tau 4R are found mainly in adults (for a review, see [1, 17]).

Although tau is mainly a neuronal protein, tau protein has been reported in several porcine tissues [18] and in rat [19]. Furthermore, its presence has been demonstrated in several cell types and tissues, including pancreatic acinar cells [20], denervated rat muscles [21], monocytes [22], testicular spermatid [23], HeLa cells as well as in non-transformed fibroblasts and lymphocytes [24, 25], skin fibroblasts [26], hepatoma cell line [25], and human prostate cancer cell [25, 27]. Tau has been also detected in muscle fibers in diverse muscle disorders [28] and in the enteric nervous system [29]. Moreover, its expression level in several human cancers has been related to resistance to anti-mitotic treatments [30, 31], although tau protein is increased in less aggressive gliomas [32].

Despite that, few attention has been paid to tau distribution in peripheral tissues and the functional significance of tau in non-neural tissues in non-pathological situations is largely unknown. Here, we have analyzed that function in kidney by using tau knockout mice generated by integrating GFP-encoding cDNA into exon 1 of MAPT [33] on a pure C57Bl/6 background. The data from kidney with tau staining show its important role in glomerular/podocyte metabolism.

## Results

### Endogenous tau promoter expression in Tau^GFP/GFP^ mice

**Fig. 1:**
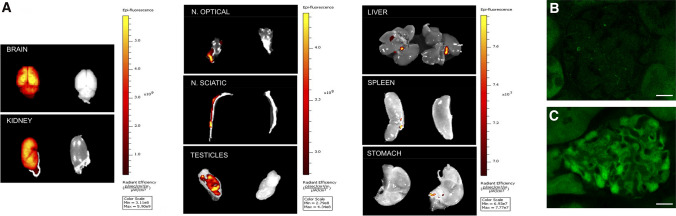
Detection of GFP in kidney of Tau^GFP/GFP^ mice. **A** Ex vivo IVIS images of organs from wild-type and Tau^GFP/GFP^ mice. The spectrum gradient bar corresponds to the fluorescence intensity unit/sec/cm^2^ is shown on the right of each organ. Fluorescence microscopy image of GFP protein in the glomerulus of wild-type (**B**) and Tau^GFP/GFP^ mice (**C**). Bar 20 μm

**Fig. 2:**
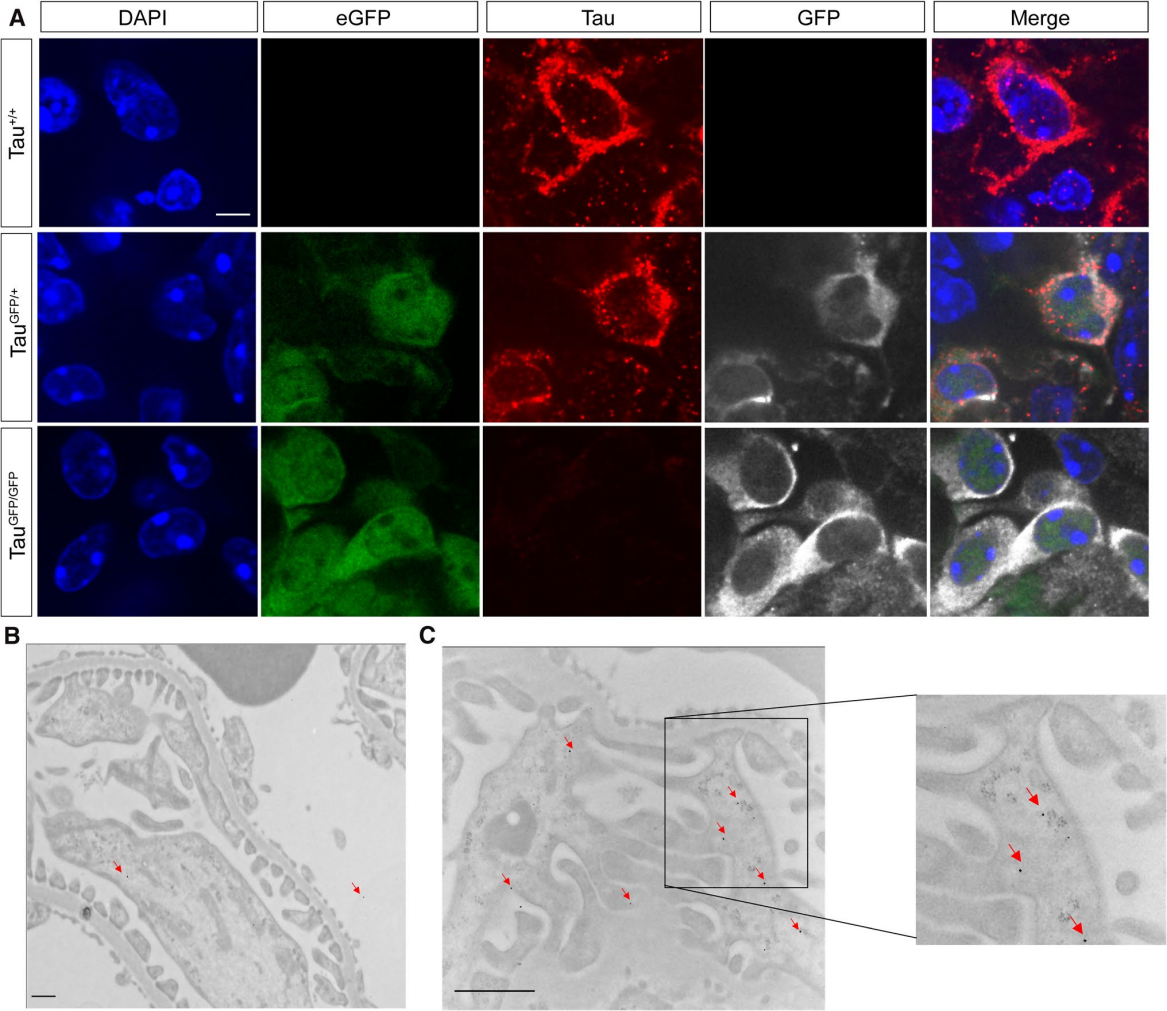
Colocalization of GFP and tau proteins in renal podocytes. **A** The localization of GFP was determined by direct fluorescence (green), indirect immunofluorescence with monoclonal antibody anti-GFP (white) and anti-total Tau (red) in wild-type (Tau^+/+^), Tau^GFP/+^, and Tau^GFP/GFP^ mice. DAPI staining is shown. Bar 20 μm. Electron micrographs of wild-type podocytes immunolabeled in the absence of primary antibody (**B**) or with antibody anti-total Tau (**C**). Ultrathin sections were labeled with 10-nm protein A-gold (red arrows). Bars 1 μm

**Fig. 3:**
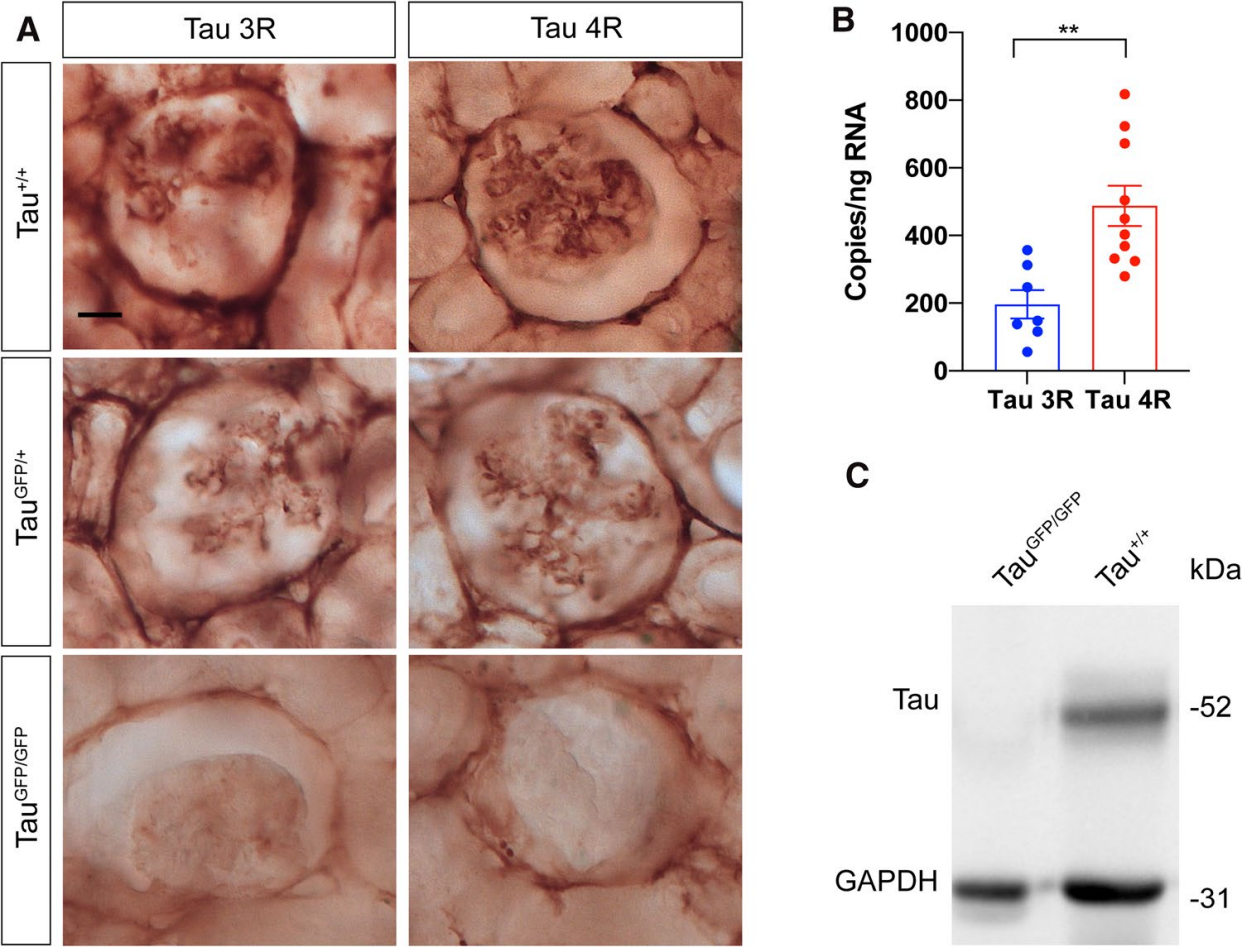
Tau protein isoforms expresses in renal podocytes. **A** Immunostaining with Tau3R and Tau4R specific anti-tau antibodies in the glomerulus of wild-type (Tau^+/+^), Tau^GFP/+^, and Tau^GFP/GFP^ mice. Bars 20 μm. **B** The absolute quantification of mRNA expression level (copies of transcript per ng total RNA) of Tau3R (*n* = 7) and Tau4R (*n* = 10) in kidney samples from wild-type mice. Results represent mean ± SEM. **P < 0.01 using unpaired Student’s *t* test. **C** The presence of tau in the total kidney protein extract was determined by Western blot using anti-total Tau antibody. As protein loading control GAPDH was used. Molecular weight is shown on the right

We investigated tau promoter expression using GFP fluorescent imaging with the IVIS Lumina II system, a rapid ex vivo whole-organ imaging system for determining GFP expression, in Tau^GFP/GFP^ mice **(**animals with two GFP copies that are knockout mice for the microtubule-associated protein tau (MAPT) gene due to insertion of the transgene GFP). We performed a side-by-side comparison of different organs from wild-type (Tau^+/+^) and Tau^GFP/GFP^ mice. Figure 1A shows the greatest fluorescence intensity in the brain, testes (as has been previously described [19, 23]), optic nerve, sciatic nerve, and kidney, while no signal was observed in spleen, liver, or stomach of Tau^GFP/GFP^ mice. This strong signal in the kidney suggests that endogenous tau promoter is expressed in this organ. Autofluorescence of GFP reporter avoids the need for antibodies for its visualization. Thus, direct observation of 50 μm slices by confocal laser scanning microscopy demonstrated that GFP was present in glomeruli and mainly in podocytes and absent in wild-type mice (Fig. 1B–C). To confirm tau expression in podocytes, we analyzed colocalization of GFP and tau protein using specific antibodies in renal sections from Tau^GFP/+^ and Tau^GFP/GFP^ mice. Immunofluorescence and confocal microscopy analysis showed that both proteins colocalize, being the staining mainly in podocytes (Fig. 2A). This was confirmed in Tau^+/+^ samples as colocalization of tau protein with nephrin, a glomerular protein marker [36], could be observed (Supplemental Fig. 1). Then, we used post-embedding immunogold electron microscopy to examine tau localization in podocytes (Fig. 2B, C). We found a number of gold particles mainly in the primary process although some gold particles could be found in foot process as well (Fig. 2B). Then, we performed immunostainings with isoform specific antibodies to know which spliced tau isoforms are present in these cells. Figure 3A shows Tau 3R and 4R antibodies labeling in wild-type (Tau^+/+^), Tau^GFP/+^ and Tau^GFP/GFP^ mice. This consisted in a pronounced labeling with Tau 4R antibody and a modest diaminobenzidine precipitation with Tau 3R antibody. These signals disappeared in Tau^GFP/GFP^ mice. These results were also validated by RT-qPCR using renal mRNA. Exon 10 is spliced out in Tau isoforms with three repeats (Tau 3R) and we confirmed the increased inclusion of *MAPT* exon 10 in renal samples, obtaining three times more tau4R than 3R copies (Fig. 3B). The presence of tau was further confirmed by immunoblot and we found a main band of apparent molecular weight of 52 kDa (Fig. 3C).

### Glomeruli show a more dynamic microtubule cytoskeleton in the absence of tau

**Fig. 4:**
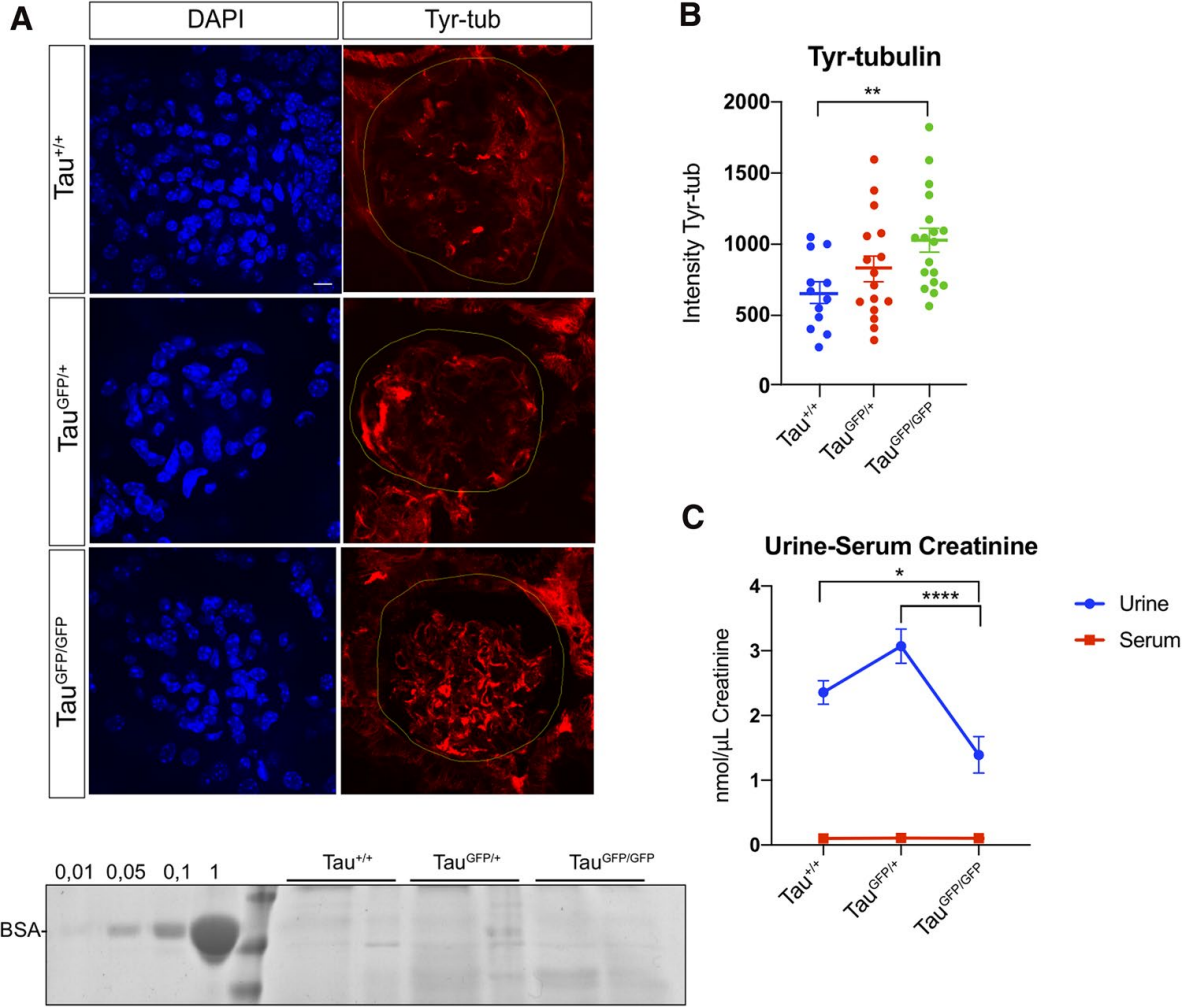
Distribution of dynamic microtubules in Tau^+/+^, Tau^GFP/+^, and Tau^GFP/GFP^ mice and kidney function. **A** Representative confocal fluorescence images showing the distribution of Tyr-α-tubulin in glomeruli (delineated in yellow) from wild-type (Tau^+/+^), Tau^GFP/+^, and Tau^GFP/GFP^ mice. DAPI staining is shown on the left. Bar 20 μm. **B** Quantitative fluorescence measurements of Tyr-α-tubulin immunolabeling in preparations. Measurements were performed in equivalent regions to those described before. Data are represented as mean ± SEM (*n* = 4, 5 glomeruli per mice). **P < 0.01 using unpaired student’s *t* test. **C** Urinary creatinine and creatinine blood levels in wild-type (Tau^+/+^), Tau^GFP/+^, and Tau^GFP/GFP^ mice. **D** Male urine samples (20 μL) were collected and were subjected to SDS-PAGE followed by Coomassie Brilliant Blue staining. BSA (0.01–1 μg) served as a standard control

Tau is a cytoskeletal protein that in neurons is involved mainly in microtubule stabilization. To analyze microtubule dynamic, we have examined levels of tyrosinated (Tyr)-α-tubulin. This post-translational mark can be used as an indirect assay of microtubule dynamics. The C-terminus of alpha-tubulin undergoes a reversible post-translational tyrosination/detyrosination process and Tyr-α-tubulin is especially enriched in the more dynamic microtubules [34–36]. Tyr-α-tubulin labeling is clearly altered in Tau^GFP/GFP^ glomeruli; thus, an increase is evident (Fig. 4A). Quantitative measurements confirmed these observations (Fig. 4B) and revealed that the balance between dynamic and stable microtubule is significantly altered in Tau^GFP/GFP^ glomeruli. An intermediate situation was observed in Tau^GFP/+^ mice.

### Renal function in Tau^GFP/GFP^ mice

**Fig. 5:**
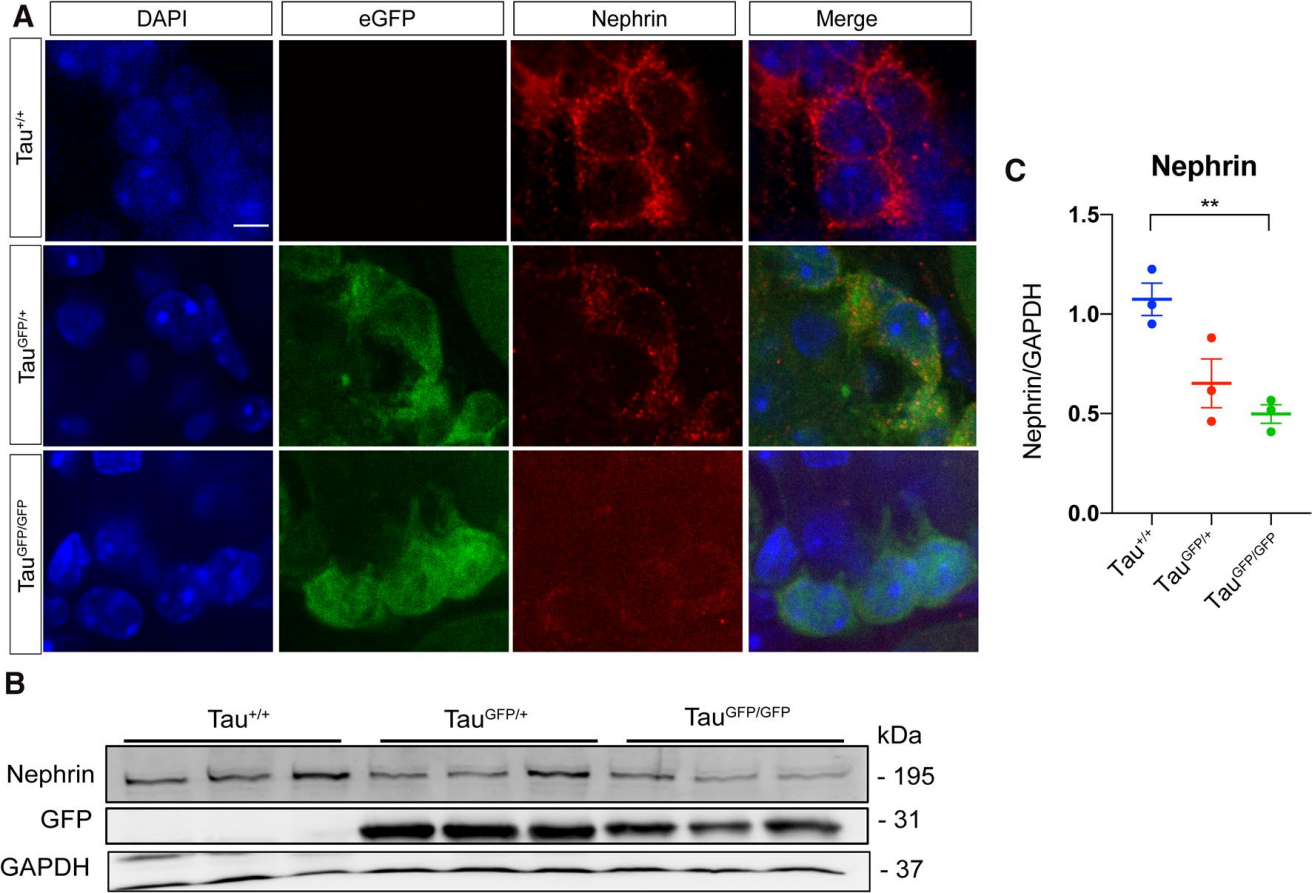
Nephrin immunostaining in Tau^GFP/GFP^ mice. **A** Expression of nephrin is reduced in the glomeruli of Tau^GFP/+^ and Tau^GFP/GFP^ mice. Representative confocal fluorescence images showing the distribution of nephrin and GFP in podocytes. DAPI staining is shown on the left. Bar 10 μm. **B** Representative Western blots of nephrin in kidney protein lysates of wild-type (Tau^+/+^), Tau^GFP/+^, and Tau^GFP/GFP^ mice. GFP levels are also shown. As protein loading controls GAPDH was used. Molecular weight is shown on the right. **C** Quantification of the expression level of nephrin shown in panel B reveals that the expression of nephrin is significantly lower in protein lysates from Tau^GFP/GFP^ mice. Bars show mean ± SEM. **P < 0.01, Student’s *t* test

To analyze if these more dynamic microtubules affect kidney function, serum creatinine and creatinine clearance were measured. Figure 4C shows that kidney function was impaired in Tau^GFP/GFP^ mice, with a reduction in urine levels compared with Tau^+/+^ littermates. Interestingly, the presence of one copy of tau protein in Tau^GFP/+^ mice seems to be sufficient for normal creatinine clearance. That decrease does not correlate with a serum creatinine increase, suggesting that kidney function is not seriously altered. However, it has been observed that one-year-old tau knockout strains show muscle weakness [37, 38]. Thus, it is possible that altered creatinine levels observed in Tau^GFP/GFP^ mice could be explained taking into account that muscle weakness. Coomassie stain of urinary proteins of 6-month-old males reveals the absence of proteinuria in any of the genotypes analyzed (Fig. 4D). Then, we focused on the expression of nephrin, one of the most important filtration barrier-associated molecules in kidney [39]. Expression of nephrin was reduced about a 50% in the glomeruli of Tau^GFP/GFP^ as shown by Western blot and immunofluorescence (Fig. 5A–C).

### Glomerular morphology in Tau^GFP/GFP^ mice

**Fig. 6:**
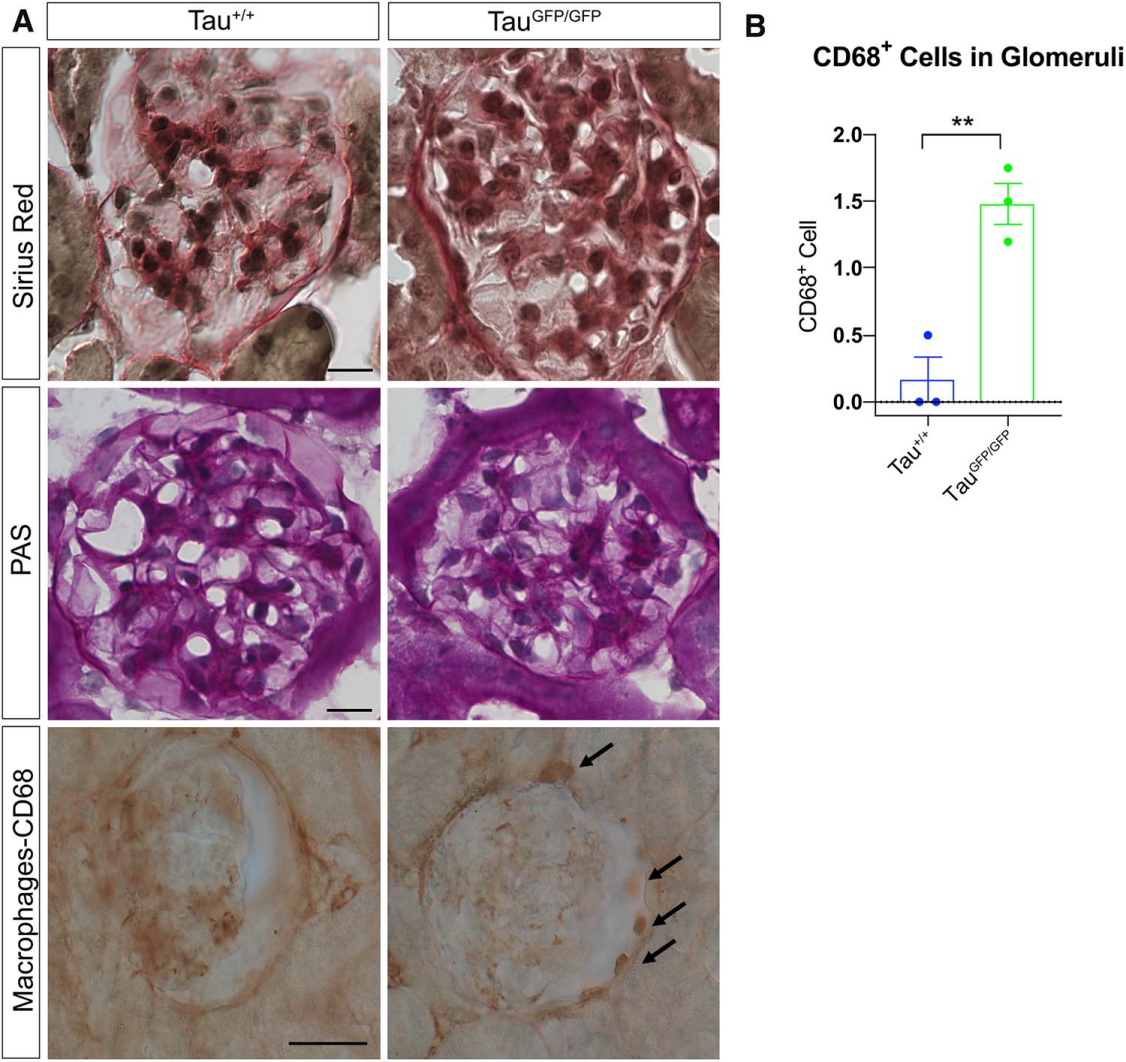
Glomerular histologic analysis and macrophage infiltration in Tau^GFP/GFP^ mice. **A** Representative images of Sirius red and periodic acid–Schiff stainings. On the left column wild-type (Tau^+/+^) mice and Tau^GFP/GFP^ samples on the right. Bar 50 μm. In bottom row, macrophages in CD68-stained sections in Tau^+/+^ and Tau^GFP/GFP^ glomeruli. Bar 25 μm. **B** Quantification of number CD68-positive infiltrating macrophages per glomerulus. Bars show mean ± SEM (*n* = 3, 5 glomeruli per mice). **P < 0.01, Student’s *t* test

Figure 6 shows Sirius red and PAS staining, stains commonly used for the histologic assessment of renal glomerular evaluation. Both techniques reveal histologically normal glomeruli in Tau^+/+^ and Tau^GFP/GFP^ mice; however, given the limitations inherent with these methods [40], we decided to perform CD68 staining, a marker of macrophages, to analyze glomerular damage (Fig. 6B). We observed an increase of infiltrate macrophages mainly inside Bowman’s space, something that was rarely observed in wild-type mice. CD68-positive cells indicated the infiltration of macrophages, a sign of inflammation, and a common feature of most human chronic kidney diseases.

**Fig. 7:**
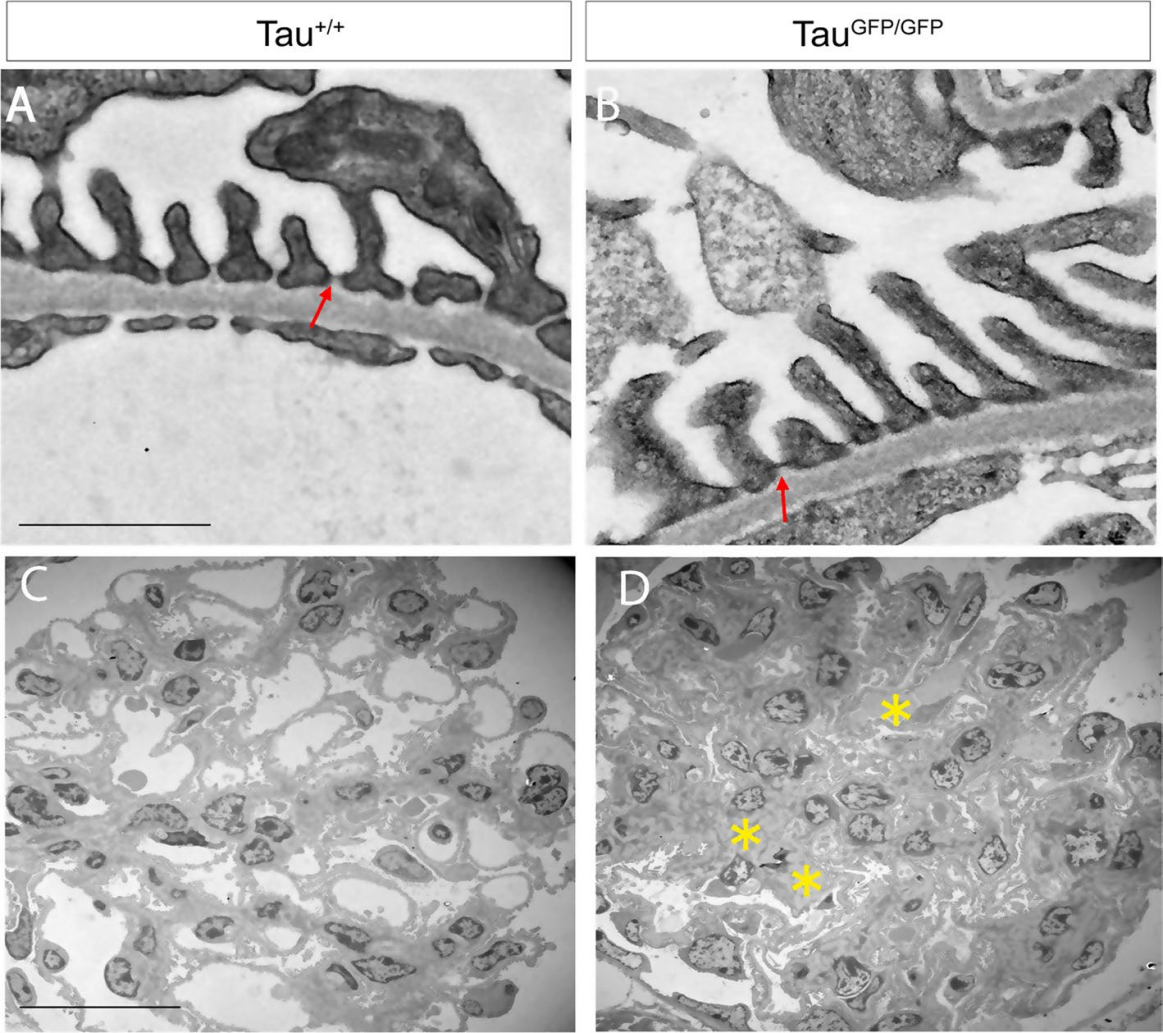
Electron microscopic photographs of a glomerulus from Tau^+/+^ and Tau^GFP/GFP^ mice. **A**, **B** Representative images of transmission electron microscopy for Tau^+/+^ (wild-type) and Tau^GFP/GFP^ glomeruli showing glomerular basement membrane, foot process, and fenestrated endothelial cells. Bars 1 μm. Red arrows = Slit diaphragms. **C**, **D** Representative images of glomeruli from Tau^+/+^ and Tau^GFP/GFP^ at low magnification. *Extracellular matrix. Bars 20 μm

Ultrastructural studies provides crucial information in many cases of glomerular diseases [41]; thus, we decided to perform an electron microscopy (EM) analysis. No main differences in endothelial cells, fenestration or glomerular basement membrane were observed (Fig. 7A, B). A frequent alteration observed in glomerular diseases is foot process effacement or fusion. In Tau^GFP/GFP^ mice podocyte foot processes line regularly the glomerular basement membrane. To our surprise, no differences in slit diaphragms were observed and they were still present despite more than 50% nephrin reduction (Fig. 7A, B, arrows). However, when we observed glomeruli by with a low magnification EM (Fig. 7C, D), an increase in the extracellular matrix was evidenced (Fig. 7D asterisks), which may eventually contribute to kidney failure.

## Discussion

This study examined the role of the microtubule-stabilizing protein tau in regulating kidney homeostasis. Our findings show that tau is expressed in renal podocytes in addition to brain. The kidney filtration barrier comprises the endothelial cells that line the blood capillaries, specialized glomerular basement membrane, and special epithelial cells called podocytes. Podocytes are highly differentiated cells that have numerous processes that interdigitate to form the final layer of the filtration barrier. Previous studies had described tau expression in renal samples from pigs [18] and rats [19]. In human, its expression has been also observed with presence of tau protein and mRNA (https://www.proteinatlas.org/ENSG00000186868-MAPT). However, few attention has been paid to tau distribution in kidney and its functional significance is largely unknown.

Our data demonstrate that the main tau isoform present in kidney is the isoform with 4-microtubule-binding domains (Tau4R) although the isoform with three domains (Tau3R) is also expressed. The Tau4R isoform promotes microtubule assembly faster than Tau3R [15]. Thus, taking into account that podocytes are highly polarized cells, Tau4R isoform seems to be important to maintain the cytoskeletal structure of these cells. In fact, our finding with Tyr-α-tubulin clearly demonstrates that the balance between dynamic and stable microtubule polymers is significantly altered in Tau^GFP/GFP^ glomeruli being the microtubular cytoskeleton more dynamic in Tau^GFP/G**F**P^ than in wild-type podocytes. In rats Tau3R has been described in renal samples [19]. Nevertheless, it should be mentioned that adult mice express mainly Tau4R isoforms in adult brain mice [42, 43] and the Tau3R isoform is expressed mainly in adult neuronal precursors [44].

Renal glomerular podocytes exhibit a highly arborized morphology similar to that present in neurons. Podocytes and neurons have thin actin-based projections (foot processes and dendritic spines, respectively) that can be considered analogous structures [45]. Further investigation is required to know the effect of tau in podocytes and its similarity or not with neuronal spines. Interestingly, tau can bind to actin [46–48] facilitating the cross-talk between microtubule and actin cytoskeletons [49].

Our finding in the present study showing that tau protein is expressed in podocytes opens up the possibilities that this protein may play pathophysiological roles in this organ. We have demonstrated that at least in Tau^GFP/GFP^ mice, and likely in situation of a loss of function of the protein, glomeruli are altered. First, expression of nephrin is reduced in the glomeruli of Tau^GFP/GFP^ mice although that reduction in nephrin protein levels does not affect slit diaphragm morphology as can be observed in electron microscopy images. Interestingly, the same was observed in mice with inducible nephrin knockdown mediated by short hairpin RNA [39]. This suggests that half of nephrin levels are enough to build kidney slits. Electron microscopy demonstrates that the Tau^GFP/GFP^ transgenic mice have larger amounts of extracellular matrix. Interstitial accumulation is a structural hallmark that correlates with a progressive kidney disease. Tau^GFP/GFP^ mice did not show problems with albuminuria although a decrease in creatinine is observed in urine. That decrease does not correlate with a serum increase, suggesting that kidney function is not seriously altered although the presence of CD68-positive cells demonstrate glomerular inflammation.

These results highlight the potential importance of tau protein in glomerular biology. We here demonstrate that absence of tau produces changes in glomerular structure suggesting compensatory mechanisms carried out by other renal microtubule-associated proteins during development. One of these proteins could be KIBRA. KIBRA colocalizes with actin and tubulin in podocytes [50]. Interestingly increasing KIBRA levels in neurons restores tau-mediated deficits in synaptic plasticity [51]. Also should be stated that these alterations have been studied in knockout mice showing absence of tau protein from birth. In this case, some of these alterations may be due to adaptations that occur during development and we do not know what happen when tau protein disappears or decrease in adults. Thus, taken into account our data, tauopathies with tau mutations as frontotemporal dementia and parkinsonism linked to chromosome 17 (FTDP-17) patients should show some type of kidney pathology. Thus, it has been described that these patients often develop urinary tract infections although it is explained as secondary medical problems associated with immobility [52, 53]. However, some patients with P301L mutation [54], N279K mutation [55], or in the + 16 mutation in the intron after exon 10 [56] report urinary incontinence.

Another consequence of our study is that therapeutic approaches for human tauopathies, focus in to evaluate the effectiveness of small interfering RNAs delivered to suppress human tau expression or other anti-tau therapies for Alzheimer’s disease currently in development [57], could potentially have unexpected off-target effects that should be considered.

In summary, the present study shows that renal podocytes express tau protein and that tau is capable of regulating microtubule dynamic. More important is that tau alters glomeruli morphology. Thus, tau protein seems to have an additional role outside the nervous system, at least in renal podocytes. Interestingly, cells have polarized morphology similar to the neurons.

## Materials and methods

### Animals and tissue processing

B6.129S4(Cg)-Mapttm1(eGFP)Klt/J mice from The Jackson Laboratory (stock 029219) with two GFP copies (here referred to as Tau^GFP/GFP^) are knockout mice for the microtubule-associated protein tau (MAPT) gene due to insertion of the transgene GFP (Supplemental Fig. 2) [33]. Tau^GFP/+^ mice with a single copy of GFP are heterozygous mice for *MAPT* gene, while the littermate Tau^+/+^ are the wild-type mice. Mice were housed in a specific pathogen-free colony facility, in accordance and handle following European and local animal care protocols. The mice used in this study were 18 months old. Animal experiments received the approval of the CBMSO’s (AECC-CBMSO-13/172) and national (PROEX 102.0/21) Ethics Committee. Mice were fully anesthetized by an intraperitoneal injection of pentobarbital (Dolethal, Vetoquinol, 60 mg/mL) and transcardially perfused with 0.9% saline.

### Ex vivo whole-organ imaging

The IVIS Lumina II system (Caliper Life Sciences) was used to determine the fluorescence intensity with appropriate excitation and emission filters. Excitation filter passband was 445–490 nm and the emission filter passband was 515–575 nm. The background filter passband was 410–440 nm. Two-dimensional images had the background signal subtracted, and image scaling was normalized by converting total radiance efficiency. Fluorescence intensity was represented by a multicolor scale ranging from red (less intense) to yellow (more intense). Signal intensity images were superimposed over grayscale reference photographs for anatomical representations.

### Immunohistochemistry and immunofluorescence

Mice whose samples were employed were fixation with 4% PFA in 0.1 N phosphate buffer (PB) for 30 h at 4 ºC. They were then washed three times in PB and placed in a 10% sucrose/ 4% agarose matrix. Sagittal kidney sections were obtained on a Leica VT1000S vibratome (50 μm). The sections were subjected to a floating immunolabeling process.

For the immunohistochemistry of 3,3′-diaminobenzidine (DAB), the VECTASTAIN Elite AB kit (Vector Laboratories) was used. The sections were washed with phosphate-buffered saline (PBS) to remove the cryoprotective solution. Subsequently, they were immersed in H_2_O_2_ to 0.33% in PBS for 45 min to block the activity of endogenous peroxidase. After three washes, sections were placed in a blocking solution (PBS with 0.5% bovine fetal serum, 0.3% Triton X-100, and 1% BSA) for 1 h and incubated overnight at 4 ºC with the corresponding primary antibody diluted in the blocking solution: tau-3R clone 8E6/C11 (mouse, 1:500, Millipore), tau-4R clone 1E1/A6 (mouse, 1:500, Millipore), and anti-CD68 (rat, 1:100, Abcam). The next day, the sections were washed three times for 10 min with PBS. They were then incubated first with the biotinylated secondary antibody for one hour, and then with the avidin-biotin-peroxidase from the kit for one hour. The developing reaction was performed using DAB for approximately 10 min. Finally, the sections were placed in slides using FluorSave (Calbiochem, Merck Millipore) as a mounting medium. Images were taken using an upright microscope Axioskop2 plus (Zeiss) coupled to DMC6200 camera (Leica).

For immunofluorescence after performing three washes with PB 0.1 N, the sections were incubated with the following primary antibodies in the blocking solution (1% BSA and 1% Triton X-100 in 0.1 N PB) at 4 ºC for 24 h: anti-tau (guinea-pig, 1:500, Synaptic systems); anti-GFP (chicken, 1:1000, Abcam); anti-a-Nephrin (guinea-pig, 1:50, Progen); and anti-tyr-tub (mouse, 1:1000, Sigma). Next, five washes were performed with this same blocking solution, and the sections were incubated with the corresponding secondary antibodies conjugated with Alexa fluorophores at 4 ºC with gentle agitation for 24 h (1:1000, Molecular Probes). Finally, three washes were performed with PB with DAPI diluted 1:5000 for 10 min, and another three additional washes were performed with PB. The sections were placed on slides using FluorSave Images that were taken using a Confocal Spinning Disk SpinSR10 attached to an IX83 inverted microscope (Olympus).

### Protein extracts and Western blots

Kidney tissue preserved at − 80 ºC was homogenized to obtain the extracts. The RIPA buffer used was 50 mM Tris–HCl pH 7.4; 150 mM NaCl; Triton X-100 1%; sodium deoxycholate 0.5%; SDS 0.1%; and phosphatase inhibitors (0.1 mM okadaic acid and 5 mM orthovanadate) plus the COMPLETE TM protease inhibitor cocktail (Roche). Next, the protein concentration of each homogenate was determined by the Bradford method [58], using the BCA test (Thermo Fisher). Finally, the SDS-PAGE buffer (250 mM Tris pH 6.8, 4% SDS, 10% glycerol, 2% β-mercaptoethanol, and 0.0006% bromophenol blue) was added to the protein extracts obtained. The extracts were boiled in a thermoblock at 100 °C for 5 min. Thirty micrograms of protein per well was loaded from each sample. The proteins were separated on 10% acrylamide/bisacrylamide gels in the presence of SDS at 120 mV for approximately 1 h. Those present in the gel were transferred to nitrocellulose membranes (Schleicher and Schuell, Keene, NH, USA) at 150 mA for 45 min, using the Bio-Rad Mini-Protean system. Subsequently, the membranes were blocked using 5% milk powder in 0.1% Tween PBS for 1 h. The membranes were then washed twice with 0.1% PBS Tween-20 (v/v) under stirring for 10 min. The membrane was stained with Ponceau dye (Ponceau 0.3% in TCA 3%) to check the transfer efficiency. Finally, they were incubated with the appropriate primary antibody overnight at 4 °C: anti-total Tau (rabbit, 1:1000, Millipore); anti-GFP (rabbit, 1:1000, Invitrogen); anti-a-Nephrin (guinea-pig, 1:500, Progen); and anti-GAPDH (rabbit, 1:1000, Cell Signaling). Protein expression was detected using the secondary antibody (1:1000) or Clean Blot (21230, Thermo Scientific), which were incubated per one hour at room temperature. After performing three 10-min washes in the wash solution, the immunoreactive proteins were detected using the ECL (Enhanced Chemiluminescence Detection System, Amersham). In the case of Nephrin, a high molecular weight protein, the polyacrylamide gel was 6% and the transfer was carried out at 4 °C at 330 mA for 1.5 h and with 10% SDS.

### Quantitative RT-PCR

Gene expression was determined by qPCR. RNA was isolated with the kit Maxwell 16 miRNA Tissue (AS1470, Promega) and the reverse transcription (RT) was performed using the iScript cDNA Synthesis Kit (1708891, Bio-Rad), according to the manufacturer’s protocols in both cases. RT-qPCR was performed using SYBR Green reagent (Applied Biosystems) and using ABI PRISM 7900HT SDS (Applied Biosystems) equipment. The reaction per well is 10 μL and contains: 0.5 μL of cDNA template per sample to bring it to 25 ng/well and bringing this volume to 4 μL with H_2_O, in addition to 5 μL of the SYBR Green PCR mix and 1 μL per oligonucleotide pair at 5 μM. The primers used were as follows: *Tau3R* forward primer: 5′-TGCCCATGCCAGACCTAAAG-3′, *Tau3R* reverse primer: 5′-TGCCCATGCCAGACCTAAAG-3′; *Tau4R* forward primer: 5′-AAGCTGGATCTTAGCAACGTC-3′, *Tau4R* reverse primer: 5′-CCGGGACGTGTTTGATATTGTC-3′. RT-qPCR amplification of genes was performed for 40 cycles of 95 °C for 1 s and 60 °C for 20 s. No amplification from the no-template control (NTC) was observed for genes of interest. To carry out the absolute quantification we used synthetics amplicons (Supplemental Table 1). Generating a standard curve with them to be able to see the number of copies that we have per well of our gene of interest. Each primer pair showed a single, sharp peak, thereby indicating that the primers amplified only 1 specific PCR product. Three technical replicates per gene were used.

### Coomassie blue staining of urinary proteins and creatinine analysis

Albuminuria was screened in male mice using SDS-PAGE followed by Coomassie Blue staining. Albumin standards were used and 20 μL of urine was run from each sample to qualitatively assess the presence of albuminuria. Urine was denatured with SDS-PAGE sample buffer and then resolved on 10% SDS-PAGE. Urinary and serum creatinine were measured using Creatinine Assay Kit (ab65340, Abcam) according to the manufacturer’s protocols.

### Electron microscopy and immunogold labeling

Mice whose samples were employed on electron microscopy (EM) were perfused with 0.9% saline followed by 4% PFA and glutaraldehyde 2% in PB. Then, kidneys were fixed with 4% PFA and glutaraldehyde 2% in PB for 2 h at room temperature and overnight at 4 °C. They were then washed three times in PB and placed in a 10% sucrose/4% agarose. Next, they were fixed with 1% osmium tetroxide in water at 4 °C for 1 h, dehydrated with ethanol, and embedded in TAAB 812 epoxy resin. Ultrathin sections of 70 nm were cut with an ultramicrotome Ultracut E (Leica) and mounted on Formvar-carbon-coated Cu/Pd 100-mesh grids; they were stained with uranyl acetate and lead citrate. A JEM1400 Flash (Jeol) electron microscope was used at 80 kV, with a Oneview 4K x4K CMOS camera (Gatan, Pleasanton, CA USA).

To carry out the post-embedding immunogold method, mice were perfused with 2% paraformaldehyde plus 0.2% glutaraldehyde in PB. 200 μm of cortical kidney slices was cryoprotected in 30% glycerol and frozen in liquid ethane in a Leica KF80. Frozen sections were immersed in 0.5% uranyl acetate in methanol at – 90 °C in a Leica AFS freeze-substitution instrument, infiltrated with Lowicryl HM 20 resin at – 45 °C, and polymerized with ultraviolet light. Thin sections of the kidney (70 nm) were incubated with glycine 0.15% in PBS for 15 min to quench free aldehyde groups, followed by blocking buffer (1% BSA, 0.2% Tween 20 and 5% FBS in TBS (Tris 30 mM, NaCl 150 mM pH 8.2) for 30 min at room temperature. Primary antibody (rabbit anti-total tau 1/10) in the same incubation buffer for 1 h followed by three times in TBS. PAG 10 nm (Protein A. Gold, Cell Microscopy Center, University Medical center Utrecht, The Netherlands) was incubated for 45 min at RT in the same buffer. Finally staining was carried out in uranyl acetate and lead citrate. A JEM1400 Flash (Jeol) electron microscope was used as described before. Controls included absence of primary antibody; controls always showed little or no gold labeling.

### Histochemistry staining

Samples were fixed in 4% paraformaldehyde (PFA) in PB and embedded in 30% sucrose for 24 h. After, the samples were embedded in OCT. Sections (10 µm) were cut with a Cryotome 230 V/50 Hz (Thermo Scientific) and mounted on superfrost glass slides.

Sirius red staining: sections were incubated in Weigert's iron hematoxylin for 8 min. Slides were then washed in running tap water for 10 min. Picro-Sirius Red solution (0.5 g Direct Red 80 + 500 mL of saturated aqueous solution of picric acid) was added for 60 min. Then acidified water at 0.5% was used for 3 min (× 2 times). Traces of water were removed by strong manual agitation and samples were dehydrated in ethanol 100% in three changes and xylol for 10 min (2× times) and later mounted.

Periodic acid Schiff (PAS) staining: Slides were placed into 0.5% periodic acid for 5 min, following this they were rinsed with distilled water. Schiff's reagent was left for 15 min. The slides were then washed in running tap water for 5 min. Mayer’s counterstain hematoxylin was added and left for 1 min. Slides were then washed with tap water. The samples were dehydrated in ethanol 96%, ethanol 100% for 5 min two times, and xylol for 10 min two times and later mounted.

### Statistical analyses

Data are expressed as mean SEM. For comparison of means between three groups, ordinary one-way ANOVA was applied. For comparisons of means between two groups, two-tailed, unpaired t tests were performed. Prism 9 (GraphPad Software, La Jolla, CA, USA) was used for statistical analyses.

## Supplementary Information

Below is the link to the electronic supplementary material.

Supplementary file1 (DOCX 750 KB)

## Data Availability

The datasets generated during the current study are available from the corresponding author on reasonable request.
