# Supplementary material for: Microtubule-associated protein tau in murine kidney: role in podocyte architecture
Source: Cell Mol Life Sci. 2022 Jan 27;79(2):97. doi: 10.1007/s00018-021-04106-z (PMC8794918; doi:10.1007/s00018-021-04106-z)
Supplement: Supplementary file 1 — Supplementary file1 (DOCX 750 KB) [file 18_2021_4106_MOESM1_ESM.docx]

**Title: Microtubule-associated protein tau in murine kidney: role in podocyte architecture.**

**Cellular and Molecular Life Sciences**

Laura Vallés-Saiz^a^, Rocio Peinado-Cahuchola^a^, Jesús Ávila^a^ and Félix Hernández^a,*^

**Affiliation:**

**^a^**Centro de Biología Molecular “Severo Ochoa”, CSIC/UAM, Universidad Autónoma de Madrid, Cantoblanco, 28049 Madrid, Spain.

***Address correspondence to**: Félix Hernández, Centro de Biología Molecular “Severo Ochoa”, CSIC/UAM, Universidad Autónoma de Madrid, Cantoblanco, 28049 Madrid, Spain; Tel.: +34 91 196 45 63; fax: +34 91 196 44 20.

E-mail address: [fhernandez@cbm.csic.es](mailto:fhernandez@cbm.csic.es)

<https://orcid.org/0000-0001-8753-8249>


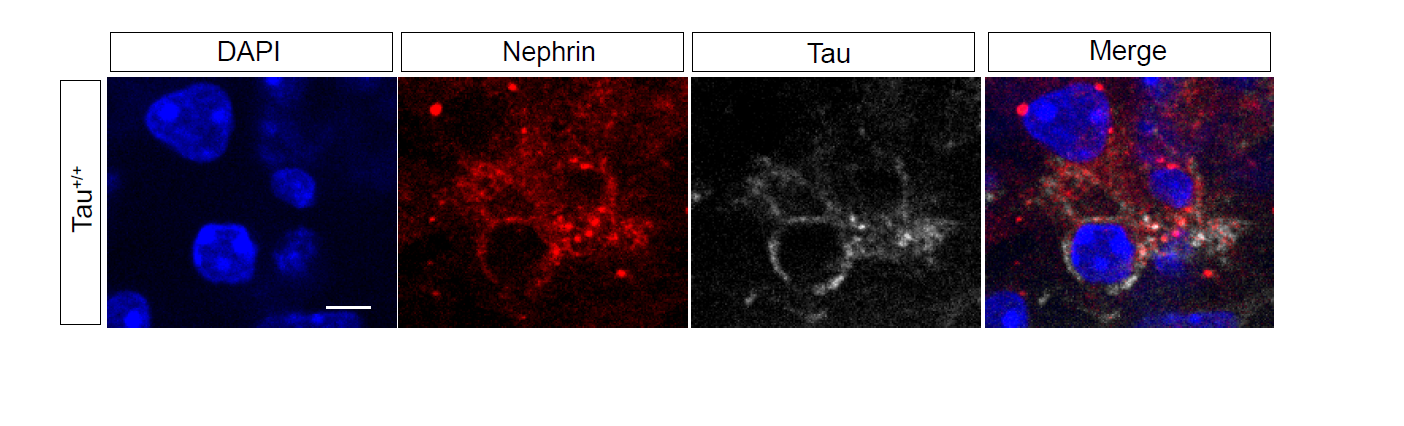


**Supplemental Fig.1. Colocalization of nephrin and tau proteins in renal podocytes.** The localization of nephrin was determined by immunofluorescence with anti-nephrin (red) and anti-total Tau (white) antibodies in Tau^+/+^ (wild-type) samples. DAPI staining is shown. Bar, 20 μm.

**Supplemental Fig.2. B6.129S4(Cg)-Mapttm1(eGFP)Klt/J mice (Tau^GFP/GFP^** **mice). (A)** Fluorescence microscopy image of GFP protein of Tau^GFP/GFP^ coronal-brain sections (left) and wilde-type (Tau^+/+^) mice (right). **(B)** Insertion in exon 1 of GFP coding sequence in *MAPT* gene showing that eGFP protein is expressed under endogenous Tau promoter.

**Supplementary Table 1.- Amplicons used to carry out the absolute quantification of Tau3R- and 4R-mRNAs.**

| GENE | AMPLICON 5’-3’ |
| --- | --- |
| Tau3R | GTGAACCACCAAAATCCGGAGAACGAAGCGGCTACAGCAGCCCCGGCTCTCCCGGAACGCCTGGCAGTCGCTCGCGCACCCCATCCCTACCAACACCGCCCACCCGGGAGCCCAAGAAGGTGGCAGTGGTCCGCACTCCCCCTAAGTCACCATCAGCTAGTAAGAGCCGCCTGCAGACTGCCCCTGTGCCCATGCCAGACCTAAAGAATGTCAGGTCGAAGATTGGCTCTACTGAGAACCTGAAGCACCAGCCAGGAGGTGGCAAGGTGCAAATAGTCTACAAGCCGGTGGACCTGAGCAAAGTGACCTCCAAGTGTGGCTCGTTAGGGAACATCCATCACAAGCCAG |
| Tau4R | TGGCAGTGGTCCGCACTCCCCCTAAGTCACCATCAGCTAGTAAGAGCCGCCTGCAGACTGCCCCTGTGCCCATGCCAGACCTAAAGAATGTCAGGTCGAAGATTGGCTCTACTGAGAACCTGAAGCACCAGCCAGGAGGTGGCAAGGTGCAGATAATTAATAAGAAGCTGGATCTTAGCAACGTCCAGTCCAAGTGTGGCTCGAAGGATAATATCAAACACGTCCCGGGTGGAGGCAGTGTGCAAATAGTCTACAAGCCGGTGGACCTGAGCAAAGTGACCTCCAAGTGTGGCTCGTTAGGGAACATCCATCACAAGCCAG |
